# Vitamin B12 status in patients of Turkish and Dutch descent with depression: a comparative cross-sectional study

**DOI:** 10.1186/1744-859X-8-18

**Published:** 2009-08-13

**Authors:** Yener Güzelcan, Peter van Loon

**Affiliations:** 1 Department of Psychiatry, Erasmus MC, University Medical Centre, Rotterdam, The Netherlands; 2 Department of Transcultural Psychiatry, Rijnmond Regional Mental Health Centre, Rotterdam, The Netherlands

## Abstract

**Background:**

Studies have shown a clear relationship between depressive disorders and vitamin B12 deficiency. Gastroenteritis and *Helicobacter pylori *infections can cause vitamin B12 deficiency. *Helicobacter pylori *infections are not uncommon among people of Turkish descent in The Netherlands.

**Aim:**

To examine the frequency of vitamin B12 deficiency in depressive patients of Turkish descent and compare it to the frequency of vitamin B12 deficiency in depressive patients of Dutch descent.

**Methods:**

The present study is a comparative cross-sectional study of 47 patients of Turkish descent and 28 of Dutch descent. The depressive disorder diagnosis and differential diagnosis were made using the Structured Clinical Interview for the Diagnostic and Statistical Manual of Mental Disorders, fourth edition text revision (SCID). The severity of the depressive symptoms was determined using the Beck Depression Inventory (BDI) and the 21-item Hamilton Depression Rating Scale (HAM-D-21). Serum baseline vitamin B6 and B12, folic acid and total serum homocysteine (tHcy) levels were measured.

**Results:**

The average ages of the patients of Turkish and Dutch descent were 40.57 and 44.75 years, respectively. There were no demonstrable differences between the serum vitamin B6, folic acid and tHcy levels in the two groups. The serum vitamin B12 levels were however clearly lower in the patients of Turkish descent than in those of Dutch descent. Vitamin B12 deficiency was however observed in 14 patients of Turkish descent and 1 of Dutch descent. This difference was significant. On the BDI, the patients of Turkish descent scored significantly higher than those of Dutch descent. Patients with vitamin B12 deficiency and those with hyperhomocysteinaemia had a significantly higher BDI score than patients with normal vitamin B12 and homocysteine levels. No relationship was observed with vitamin B12 and tHcy.

**Conclusion:**

Vitamin B12 deficiency occurs more frequently in depressive patients of Turkish than of Dutch descent. This is why it is advisable to test the vitamin B12 serum level in depressive patients of Turkish descent.

## Introduction

Various biological factors play a role in the aetiology of depression [1-3] and vitamin B12 deficiency is one such biological factor [4,5]. There is evidence of vitamin B12 deficiency in 5% to 10% of the Dutch population [6], and it is clear from the literature that poor vitamin B12 status is accompanied by an increased prevalence of depressive and other neuropsychiatric disorders [4,7-12]. In one study, 30% of clinical patients who were depressed had evidence of vitamin B12 deficiency [8]. Vitamin B12 deficiency results in hyperhomocysteinaemia and, in addition to vascular problems, this can also cause psychiatric disorders [13]. Hyperhomocysteinaemia plays a role in schizophrenia, personality disorders, obsessive-compulsive disorders, postoperative delirium, postoperative psychoses, anorexia nervosa and depression [14-16].

Vitamin B12 status is determined in part by diet [17], an optimal resorption of the consumed vitamin B12 and the presence of Gram-negative rod-shaped *Helicobacter pylori *(*H. pylori*), [18,19]. An insufficient consumption of vitamin B12 can ultimately result in vitamin B12 deficiency [17]. The presence of *H. pylori *not only plays a direct role in the vitamin B12 status, but it also impedes optimal resorption of vitamin B12 via atrophy of the abdominal mucous membrane ensuing from infection [20]. Atrophy results in an inadequate linking between the consumed vitamin B12 and intrinsic factor. It has been demonstrated in The Netherlands that *H. pylori *infections occur more frequently in patients of Turkish descent than of Dutch descent [21,22]. Consequently, this can result in vitamin B12 deficiency occurring more frequently in patients of Turkish decsent than of Dutch descent. There is no recorded data on the frequency of vitamin B12 deficiency among people of Turkish descent in The Netherlands. In this study, we examined whether there were any differences between the occurrence of vitamin B12 deficiency in patients of Turkish and of Dutch descent with depression.

## Methods

### Patients

We performed a cross-sectional study focused on inpatients and outpatients in the psychiatric ward of a general hospital (47 depressed patients of Turkish descent and 28 of Dutch descent). The patients in this study were in the age 18 to 65 age group with a depressive disorder according to the Diagnostic and Statistical Manual of Mental Disorders, fourth edition text revision (DSM-IV) classification system, and of Dutch or Turkish descent. The diagnosis and comorbid psychiatric diagnosis were made by one of the authors (YG) using the Structured Clinical Interview for the DSM-IV (SCID) [23]. All patients were included and screened after intake and before treatment. Included patients may have been taking psychopharmaceutics, but not lithium.

Patients who were excluded were known to have a vitamin B12 deficiency, were already being treated for a somatic disorder accompanying a vitamin B12 deficiency, had severe cognitive disorders or severe psychotic complaints or were severely suicidal, took vitamin supplements or medication that could result in hyperhomocysteinaemia, were dependent on alcohol or drugs or were pregnant.

The study was approved by the Medical Commission of the Reiner van Arkel Group in 's-Hertogenbosch.

## Instruments and procedures

### Psychological instrument

The diagnosis of depression was made according to the DSM-IV classification system using the SCID. The severity of the depressive symptoms was measured using the Beck Depression Inventory (BDI) [24] and the 21-item Hamilton Depression Rating Scale (HAM-D-21) [25].

### Somatic screening and assays

A general physical examination was conducted to exclude the possibility of a physical cause of the psychiatric illness. A laboratory examination was also performed that covered electrolytes, hepatic function, renal function, C-reactive protein (CRP), sedimentation, haemoglobulin, lipoprotein, serum vitamins B6, B12, folic acid and total serum homocysteine (tHcy). The blood samples were measured on a fasting basis between 8.00 AM and 10.00 AM at the hospital laboratory. Competitive electrochemiluminescence immunoassay (ECLIA) on a Modular E170 Roche Diagnostics device (Roche Diagnostic Mannheim, Germany) was used to measure the serum vitamin B12 level (cut-off 145 pmol/L). The reverse-phase high performance liquid chromatography (HPLC) method, which measures pyridoxal-5 phosphate, was used to measure the vitamin B6 level. Competitive ECLIA on a Modular E170 Roche Diagnostics device was used to measure the folic acid level. To measure the total plasma homocysteine level, the total homocysteine level was measured using reverse-phase HPLC after the protein-linked homocysteine was released using the Fa BioRad kit (Bio-Rad Quantaphase kit; Bio-Rad Clinical Division, Hercules, Calif).

### Statistical analysis

The patient features were analysed via descriptive statistics. The differences between the various subgroups at the various measuring moments and the interval and ratio data with a normal distribution were tested with the parametric Student t test. Interval and ratio data without a normal distribution and data of an ordinal measuring level was tested using the non-paramedical Wilcoxon test (two dependent measurements). The Pearson χ^2 ^statistic was used for the category-linked variables. The Pearson correlation test was used to test the correlation between clinical data and vitamins and tHcy. A *P *value < 0.05 was viewed as statistically significant. The statistical calculations were performed using the SPSS 11.5.1 software program (SPSS Inc. Chicago, IL, USA).

## Results

### Demographic and clinical data

As is clear from Table 1, the average age of patients of Turkish descent was 40.57 years (SD 8.1) and for patients of Dutch descent 44.71 years (SD 10.8). The difference was not significant (*P *value 0.74). In all, 30 (63.8%) of the patients of Turkish descent were female, as were 19 (67.8%) of the patients of Dutch descent (*P *value 0.723). The average BDI score for patients of Turkish descent was 33.57 (SD 11.57), and was 27.59 (SD 10.14) for patients of Dutch descent. Patients of Turkish descent had a relatively higher BDI score than those of Dutch descent. The difference was significant (*P *value 0.038). Patients of Turkish descent had an average score of 34.67 (SD 11.25) on the HAM-D-21, and those of Dutch descent had an average score of 31.76 (SD 7.95). The difference was not significant (*P *value 0.259).

**Table 1:** Demographic information and clinical data on patients

Demographic or clinical data	Patients of Turkish descent, n = 47 (62.66%)		Patients of Dutch descent, n = 28 (37.33%)		t Test	*P *value
Mean (SD) age, years	40.57	8.81	44.71	10.88	-1.815	0.074
Female sex, n (%)	30	63.8	19	67.8	0.126^a^	0.723
Comorbid psychiatric illness	32	68.08	10	35.71	7.462	0.006
Mean (SD) BDI (0 to 63)	33.57	11.57	27.59	10.14	2.127	0.038
Mean (SD) HAD-D-2	34.67	11.25	31.76	7.95	1.138	0.259

^a^χ^2 ^test.

A total of 32 patients of Turkish descent had 1 or 2 comorbid psychiatric disorders, as did 10 of the patients of Dutch descent. Patients of Turkish descent therefore had more comorbid psychiatric disorders (*P *value 0.006). Post-traumatic stress, panic and obsessive-compulsive disorders were the comorbid psychiatric disorders observed. Post-traumatic stress disorder was the most common comorbid disorder among both sets of patients.

### Vitamins and tHcy

#### Differences between patients of Turkish and Dutch descent

Table 2 shows that the average vitamin B6 level was 62.28 nmol/L (SD 16.18) in patients of Turkish descent and 68.96 nmol/L (SD 16.18) in those of Dutch descent. Therefore it was lower on average in patients of Turkish descent than in those of Dutch descent. The difference was not significant (0.138). There was no vitamin B6 deficiency in either of the groups.

**Table 2:** Vitamin B and homocysteine levels

	Patients of Turkish descent, n = 47 (62.66%)		Patients of Dutch descent, n = 28 (37.33)		Statistics	*P *value
Vitamin B6, mean (SD)	62.28	16.18	68.96	16.18	-1.481^a^	0.138
Vitamin B12, mean (SD)	222.87	105.0	293.71	96.33	-3,314^a^	0.001
Folic acid, mean (SD)	16.67	6.74	16.68	6.87	-0.208^a^	0.835
Homocysteine, mean (SD)	11.27	6.30	10.61	3.04	0.355^a^	0.723
Vitamin B12 deficiency, n (%)	14	29.79	1	3.70	7.219^b^	0.007
Hyperhomocysteinaemia (>15), n (%)	5	11.11	3	11.11	0.000^b^	1.000

^a^Z score; ^b^χ^2 ^test.

The average vitamin B12 level was 222.87 pmol/L (SD 105.40) in patients of Turkish descent and 293.71 pmol/L (SD 96.33) in those of Dutch descent. therefore it was lower on average in patients of Turkish descent than in those of Dutch descent. The difference was significant (*P *value = 0.001).

The average folic acid level was 16.67 nmol/L (SD 6.74) in patients of Turkish descent and 16.68 nmol/L (SD 6.68) in those of Dutch descent. Therefore it was somewhat lower on average in patients of Turkish descent than in those of Dutch descent. The difference was not significant (*P *value 0.835). There was no folic acid deficiency in either of the groups.

The average homocysteine level was 11.2 μmol/L (SD 6.30) in patients of Turkish descent and 10.61 μmol/L (SD 0.04) in those of Dutch descent. Therefore it was higher on average in patients of Turkish than in those of Dutch descent. The difference was not significant (*P *value 0.723).

No correlation was observed between the severity of the depressive symptoms and the vitamin and homocysteine levels in the blood. There was a clear negative correlation, however, with the vitamin B6, B12 and folic acid levels and homocysteine.

#### Effect of B12 deficiency

A total of 14 (29.79%) of the patients of Turkish descent and 1 (3.70%) patient of Dutch descent had vitamin B12 deficiency. The difference was significant (*P *value 0.007). The patients with vitamin B deficiency had higher BDI and HAM-D-21 scores than those with normal vitamin B12 levels. The difference was significant (0.046) as regards the BDI, but not as regards the HAM-D-21.

#### Effect of hyperhomocysteinaemia

Hyperhomocysteinaemia (Table 3) was observed in five patients of Turkish descent and three patients of Dutch descent. The difference was not significant (*P *value.1.00). The patients with hyperhomocysteinaemia had significantly higher BDI and HAM-D-21 scores than those with a normal homocysteine level in the blood. The difference in the BDI was significant (0.044), but the difference in the HAM-D-21 was not.

**Table 3:** BDI and homocysteine scores in patients with vitamin B12 deficiency and hyperhomocysteinaemia

	No vitamin B12 deficiency, mean (SD)	Vitamin B12 deficiency, mean (SD)	t Test	*P *value	No hyperhomocysteinaemia, mean (SD)	Hyperhomocysteinaemia, mean (SD)	t Test	*P *value
BDI	29.81 (10.39)	38.37 (15.3)	-2.036	0.046	29.82 (10.51)	39.14 (15.99)	-2.063	0.044
HAM-D-21	33.18 (10.59)	34.41 (8.03)	-0.38	0.0705	33.71 (10.51)	36 (8.22)	-0.587	0.559

BDI, Beck Depression Inventory; HAM-D-21, 21-item Hamilton Depression Rating Scale.

## Discussion

Vitamin B12 levels were clearly lower in patients of Turkish descent than in those of Dutch descent. A total of 14 of the patients of Turkish descent had a vitamin B12 deficiency, as did 1 patient of Dutch descent. The patients who had a vitamin B12 deficiency had higher BDI scores than those who did not. Atrophic gastritis is known to be one of the reasons for vitamin B12 deficiency. Infection with *H. pylori *is one of the risk factors for vitamin B12 deficiency. Almost 82% of people of Turkish descent in The Netherlands are infected with *H. pylori *[22,26]. The same study shows that 4.85% of the patients of Turkish descent have atrophic gastritis, as do 0% of the patients of Dutch descent. Sizeable levels of vitamin B12 deficiency are observed in patients of Turkish descent. Vitamin B12 deficiency can be correlated with depressive complaints. Earlier studies have demonstrated the correlation between vitamin B12 deficiency and neuropsychiatric disorders, such as depression [4,5]. The underlying causes of vitamin B12 deficiency were not further examined in this study. Vitamin B12 deficiency can be linked to eating habits, hereditary factors or other somatic causes. This has potential for follow-up in a further study and might well provide greater insight into the aetiology of vitamin B12 deficiency in this group of patients. The study by Miscoulon *et al*. [27] discusses 213 depressive patients treated with fluoxetine 20 mg/day. The effect of plasma folic acid and vitamin B12 status on the treatment effect of fluoxetine was examined. Folic acid and vitamin B12 status do not appear to be predictors of recidivism in depressive patients. The treatment with fluoxetine was less effective if there was evidence of a low plasma vitamin B12 level. Hintikka *et al*. [28] demonstrated in a naturalistic prospective follow-up study that depressive patients with high vitamin B12 serum levels respond better to treatment for depressive complaints than patients with lower vitamin B12 serum levels.

In another study [9], no correlation with vitamin B12 deficiency was observed with respect to depressive symptoms in the general patient population. In two studies, the effect of vitamin B12 supplementation on depressive symptoms was not examined [29,30]. This would be useful to examine in future research. Earlier studies have shown that remedying a vitamin B12 deficiency has a positive effect on depressive symptoms [31]. Depressive and neuropsychological complaints can be caused by various mechanisms in patients with a vitamin B12 deficiency [32-34]. One of the explanations is an increased tHcy level in patients with vitamin B12 deficiency. In this study, there was a negative correlation between the tHcy level and the vitamin B12 level. This study did not focus on the differences between the various generations of Turkish descent. Researching the differences between the various generations could produce data on aetiological factors.

Vitamin B12 deficiency is more common among patients of Turkish than of Dutch descent. This is why it is important to conduct a standard test of the vitamin B12 serum level in this group of patients.

## Competing interests

The authors declare that they have no competing interests.

## Authors' contributions

YG carried out the vitamin B12 status in patients of Turkish and Dutch with depression study, participated in the sequence alignment and drafted the manuscript. PvL participated in the sequence alignment and drafted the manuscript. All authors read and approved the final manuscript.
